# Temporomandibular Joint Herniation into the External Auditory Canal

**DOI:** 10.5334/jbsr.3104

**Published:** 2023-06-05

**Authors:** Daan Arie G. Coppens, Jan Casselman

**Affiliations:** 1 KU Leuven, BE; 2 AZ Sint-Jan Bruges, BE

**Keywords:** temporomandibulair joint hernation, external auditory canal, temporal bone CT scan, foramen of Huschke

## Abstract

**Teaching point:** Always ask a patient to open and close their mouth when you see a mass protruding into the external auditory canal, as to not miss this rare anomaly.

## Case History

**Figure 1A:**
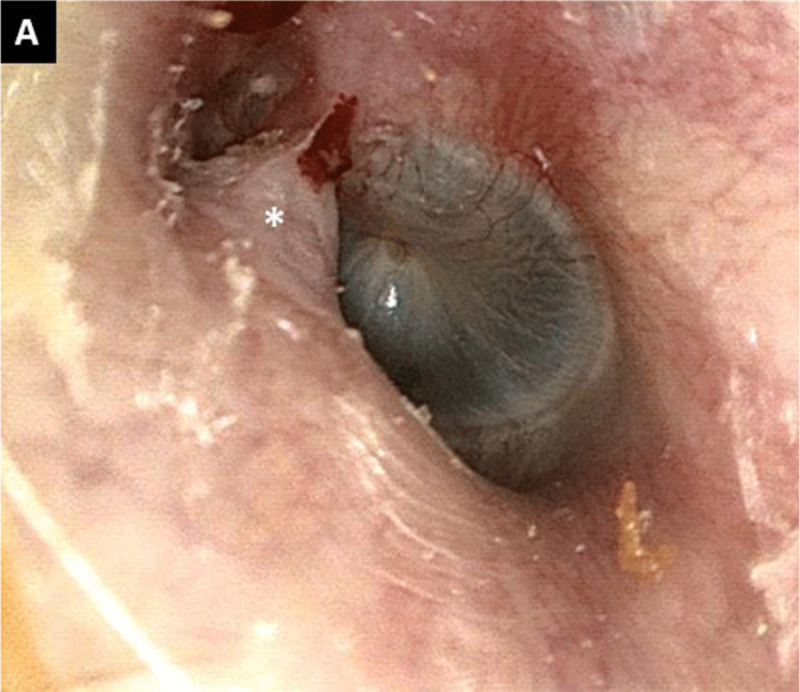


**Figure 2B-C:**
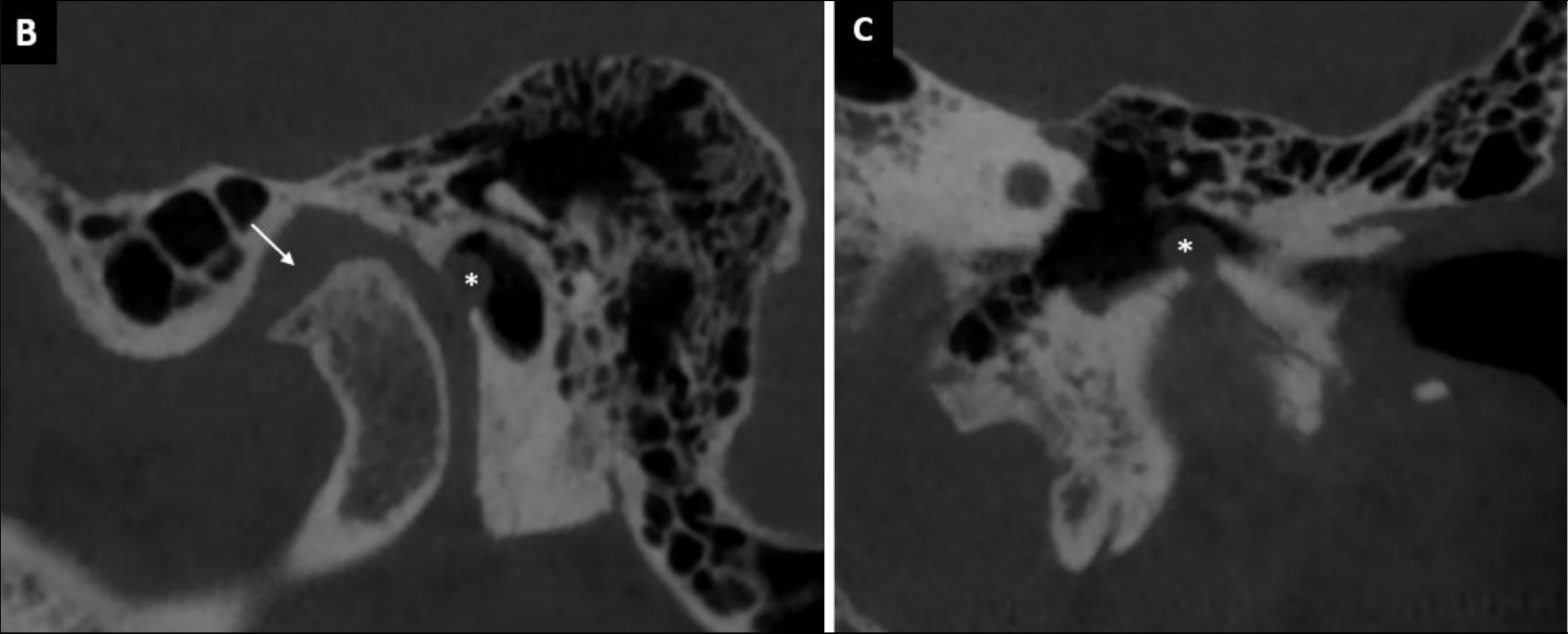


A 66-year old man presented with an intermittent foreign body sensation and a mild pressure feeling in the left ear for the last five years. He didn’t perceive any hearing loss or other otologic complaints. Otomicroscopy revealed a mass at the antero-superior portion of the bony external auditory canal (EAC), with an adhesion to the tympanic membrane (Figure 1A). When he closed and opened his mouth, the mass could be seen to appear and disappear into the EAC (Movie). A temporal bone computed tomography (CT) scan was performed and showed a herniation of the capsule of the temporomandibulair joint (TMJ) into the EAC through the foramen of Huschke (foramen tympanicum) (Figure 2B–C). It also showed destruction of the head of the condylar process of the mandible, suggesting degenerative joint disease as possible underlying cause of the herniation. Conservative management was recommended, and the patient was asked to take analgesics if needed, and to reduce his left TMJ load by avoiding heavy or excessive chewing on this side. After six weeks, the symptoms did not disappear; however, the patient reported a moderate improvement of both symptoms.

## Comments

Temporomandibular joint herniation into the EAC is a very rare condition and since it is mostly case reported, the exact incidence is unknown. A review [1] from 51 previous cases on the cause showed four separate categories being traumatic (7.8%), inflammatory (11.8%), iatrogenic (17.6%) and spontaneous herniation (62.7%). One should consider a TMJ herniation if patients complain of symptoms when chewing or eating. Further, symptoms seem to be rather nonspecific, but can vary from otalgia and otorrhea to a clicking tinnitus and hearing loss, depending on the underlying cause of the herniation. A temporal bone CT scan can confirm the diagnosis [1]. Treatment options depend on the severity of the perceived symptoms. Conservative care consists of measures such as analgesics, muscle relaxants, and chewing food on the side opposite to the herniation. Several options exist for surgical management. Resection of the mass can be done with or without wall reconstruction via preauricular, endaural, or transcanal approach.
